# Evaluation of the Dislodgement Resistance of Traditional Adhesive Posterior Bridges Compared With Adhesive Posterior Bridges Prepared With Standard and Modified Slot-Back Dummies: An In Vitro Study

**DOI:** 10.7759/cureus.71087

**Published:** 2024-10-08

**Authors:** Waseem Alkateb, Hassan A Husein, Issam Jamous

**Affiliations:** 1 Fixed Prosthodontics, Damascus University, Damascus, SYR

**Keywords:** adhesive bridges, dislodgement resistance, minimal preparation bridges, modified slot-back dummy, slot-back dummy

## Abstract

Background

Bridges with minimal preparation have received great acceptance in recent years. Since their first appearance, they have undergone many types of modifications and improvements.

Aim

This study aimed to compare three types of minimal preparation bridges in terms of force required for dislodgement and the type of deformation incurred for each of the abutments and prostheses.

Materials and methods

The research sample consisted of 36 adhesive bridges divided into three equal groups. The first group contained traditional adhesive bridges prepared from the proximal and lingual surfaces with a thickness of 1 mm, the second group contained adhesive bridges with standard slot-back dummies, and the third group contained adhesive bridges with modified slot-back dummies. Each bridge underwent a pressure test, which was directed from the buccal toward the lingual side and was inclined to the horizontal plane at an angle of 45°. A one-way analysis of variance (ANOVA) test was conducted with a significance level of 0.05.

Results

The average dislodgement resistance value in the traditional adhesive bridges group was 480,858 N, with no statistically significant difference from the standard slot-back dummy group (p = 1), for which the average dislodgement resistance value was 486,050 N. The average dislodgement resistance value in the modified slot-back dummy group was 746,733 N, with a statistically significant difference compared with other study groups (p < 0.05).

Conclusion

The adhesive bridge with the modified slot-back dummy showed higher dislodgement resistance compared to the traditional adhesive bridge and the adhesive bridge with the slot-back dummy.

## Introduction

Minimal preparation bridges are designed to replace missing teeth without the need for extensive preparation of the tooth tissue. This type of bridge is fixed, preserves tooth tissue, and is both easy to manufacture and cost-effective. It is also a reversible treatment method. However, there are some drawbacks associated with adhesive bridges, such as reduced stability and dislocation. Adhesive bridges are often less stable than traditional bridges and can dislodge more easily, especially when exposed to high mechanical stress or large biting forces. Furthermore, because of this limited durability and tolerance to mechanical forces, adhesive bridges are less able to withstand significant biting forces, making them more prone to fracture and/or dislocation [1].

At the beginning of using this type of bridge, no preparation was done as the abutments were only cleaned with the ultrasonic devices, and the casted splint was directly adhered to the teeth. However, in the next design, vertical and horizontal grooves were prepared on the lingual surface. This preparation did not penetrate the dentin-enamel junction and was kept within the enamel thickness. This design was assumed to provide mechanical support to reduce the amount of stress forces transmitted to the bonded area, and it consisted of an acrylic dummy only [2]. Later, casted perforated adhesive restorations were used on the posterior teeth [3].

Pins were then used to increase the stability of the posterior teeth by preparing the occlusal surfaces. As a result of the increased stability of this method, the researchers suggested reducing the preparation of the lingual surface. They also suggested applying this method in cases of short crowns, for which the traditional shape does not provide adequate stability [4].

The traditional form of adhesive restorations was modified by preparing the four axial surfaces of the tooth with a supra-gingival finish line in a semi-shoulder form [5], as well as a finish line in the form of a knife edge from the occlusal side, approximately 0.5 mm away from the occlusal contact points and with the opposing teeth in the gingival direction. This design was called the "sleeve" or "full-surrounded" design.

After that, the improvements were limited to refining the preparation methods of the previously proposed designs. It was proposed to determine the outer border of the preparation design on gypsum casts in order to make adhesive restorations on the posterior teeth using the surveyor to form a reference and guide during the clinical preparation. Several colors were used for planning, and by changing the inclination of the casts, the best planning could be maximized to ensure a suitable insertion line without over-preparing the teeth [6]. Many modifications were made to the degree of convergence of the preparation walls, as stability decreases with increasing convergence regardless of the type of cement used for bonding [7]. After reviewing the improvements in the design and preparation of adhesive bridges, we found that the preparation method, the choice of choosing the appropriate design for each case, and the implementation of the preparation accurately are essential and decisive factors in determining the durability, success, and survival rate of these bridges.

Therefore, this study aimed to compare traditional adhesive bridges against adhesive bridges prepared with standard and modified slot-back dummies in terms of the amount of force required for their dislodgement of each type and the type of deformation that occurs to each of their abutments and prostheses after their dislodgement.

## Materials and methods

Study design

**Figure 1:**
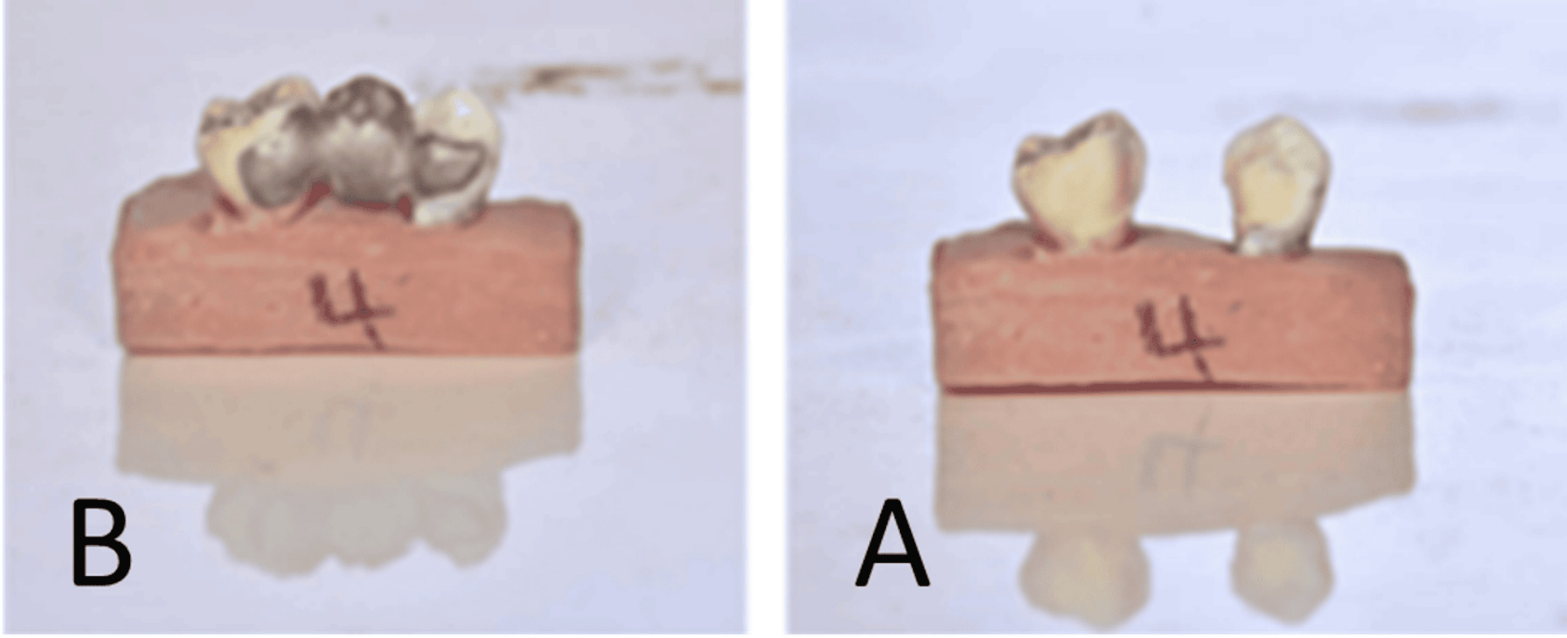
The first group: traditional adhesive bridge A: Preparation of traditional adhesive bridge. B: The bridge after cementation.

This study was a comparative in vitro study of three groups. In the first group, the second premolar was replaced by a traditional adhesive bridge prepared from the lingual and distal surfaces with a thickness of 1 mm (Figure 1).

**Figure 2:**
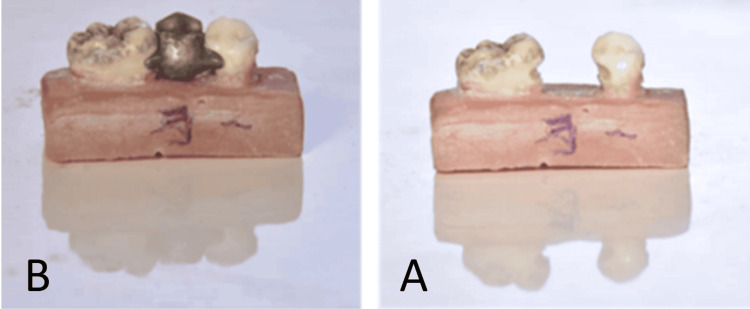
The second group: adhesive bridges with standard slot-back dummy A: Preparation of adhesive bridge with a standard slot-back dummy. B: The bridge after cementation.

The second group included adhesive bridges with standard slot-back dummies that were completely vertical without angulation (Figure 2).

**Figure 3:**
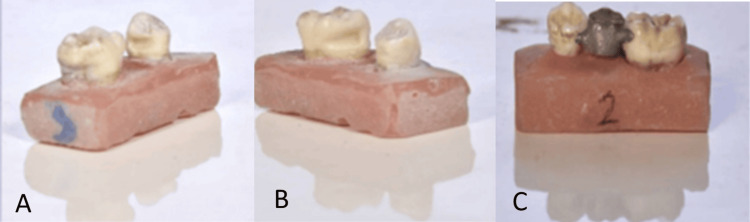
The third group: adhesive bridges with modified slot-back dummy at 30° of angulation A and B: Preparation of adhesive bridge with a modified slot-back dummy. C: The bridge after cementation.

The third group included adhesive bridges with slot-back dummies and 30° of angulation (Figure 3).

Groups were compared in terms of how well they dislodged from the teeth and the force required for dislodgement when subjected to a 45° oblique vestibular force.

Method

Thirty-six extracted mandibular first premolars and 36 extracted mandibular first molars were collected, intact from the proximal surfaces, disinfected by placing them in a 0.5% chloramine T solution at 37°C, and then immersed in sterile serum until preparation.

The bridges were formed by casting each premolar and molar into an acrylic base, maintaining a distance between them equal to the mesiodistal width of the mandibular second premolar to reproduce, so that it would be similar to its position in the oral cavity. The research sample was then divided into three groups, each containing 12 bridges. The first group used traditional adhesive bridges prepared from the proximal and lingual sides with a thickness of 1 mm. The second group used adhesive bridges with slot-back dummies, as explained below. The third group used adhesive bridges with slot-back dummies with a modification to the preparation axis, which was inclined from the buccal to the lingual surfaces in the gingival direction at an angle of 30° so that the entrance was from the lingual side.

After preparing the samples, each bridge was subjected to a compressive force from the buccal side toward the lingual side and tilted to the horizontal level at an angle of 45°.

Principle of preparing the standard slot-back dummy

The new design explained below is called the "slot-back dummy" or "slot-back bridge." The stability of this dummy depends on the principle of mechanical retention, by preparing grooves, which serve as rails, in the abutments on the proximal surfaces adjacent to the edentoula. Within each groove, there was a wing for the prosthetic dummy, where the dummy had mesial and lateral wings.

To prepare the slot-back dummy, the abutment must be intact from the proximal side adjacent to the ridge, and the clinical length of the abutments must be sufficient.

Preparation of the first lower molar

**Figure 4:**
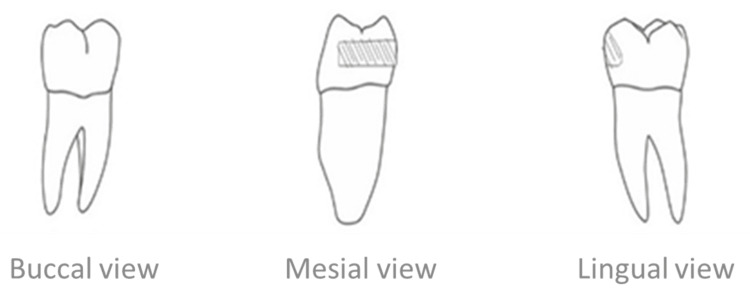
Delineation of the preparation of the first lower molar

The preparation of the first lower molar was done using a 2-mm fissure bur. The entrance of the preparation was axio-lingual to the buccal surface without penetrating it. The entry point was horizontal and parallel to the greater periphery of the tooth so that the lower wall of the preparation was at the level of the greater periphery (Figure 4).

During preparation, the gingival wall was initially a straight horizontal line and then rotated close to the pulpal wall for the full thickness of the bur (i.e., at least 2 mm). Since the axial wall was inclined to occlusal from the greater circumference of the tooth, the occlusal wall was shorter than the gingival. This resulted in unsupported enamel on the occlusal surface, which was beveled to have at least 1 mm of thickness of the remaining enamel.

Preparation of the lower first premolar

The preparation paralleled that of the molar but not necessarily at the same gingival-occlusal level. The starting point of the preparation was also at the lingual surface, taking into account that the large periphery of the premolar is very close to the occlusal surface, so the preparation would not be in the molar but rather would be estimated so that it would be at the farthest point from the pulp horn.

**Figure 5:**
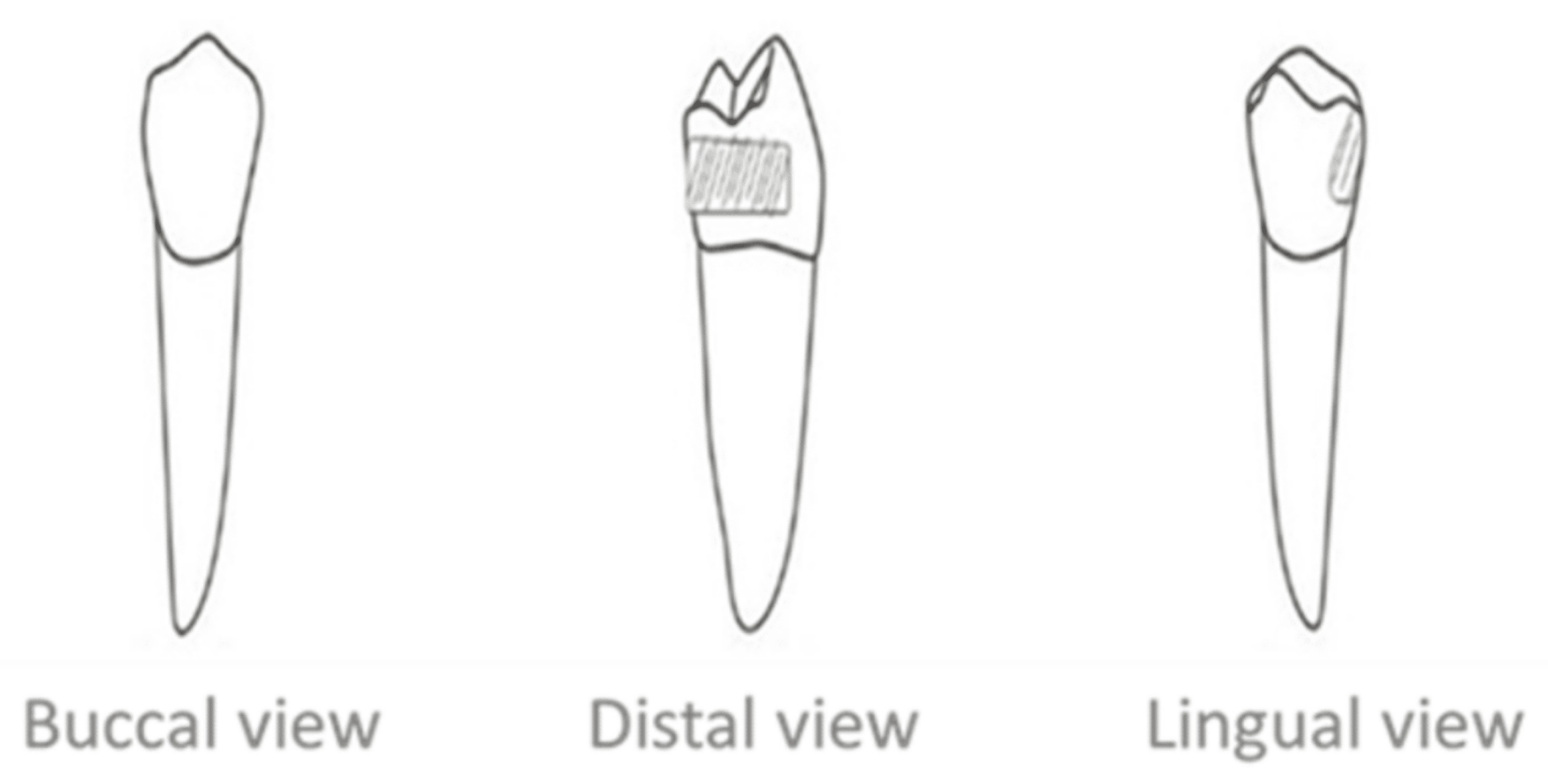
Delineation of the preparation of the lower first premolar

The preparation was done with the same fissure bur so that the floor of the cavity (i.e., the gingival surface of the preparation) would be straight at the edge of the tooth as in the molar and then rotated according to the shape of the bur at the pulp wall of the preparation. In addition to beveling the unsupported enamel, the occlusal wall was also beveled (Figure 5).

**Figure 6:**
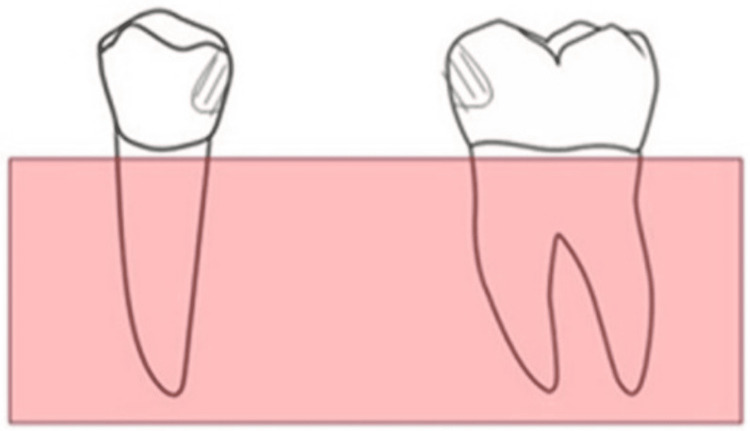
Delineation of the preparation of the premolar and the molar from the lingual side

After completing the preparation of the grooves, a clear insertion line was ensured so that the two cavities diverged from the buccal toward the lingual surface (Figure 6).

Preparation of the modified slot-back dummy

An acrylic base was made at an angle of 30°, with the same preparation as for the slot-back dummy. After placing the sample on the base horizontally, the preparation was tilted at an angle of 30° from lingual to buccal and from occlusal to gingival.

The dummy

Since the insertion axis is lingual, this imposes a specific shape for the dummy such that it touches the saddle with a point, meaning it must be oval or conical to ensure aesthetic aspects. If the aesthetic aspect is not a priority, it can be a hygienic dummy.

Impression

Impressions were taken using the CADstar Ultra extraoral scanner (Bischofshofen, Austria), bridges were designed using a computer, and metal was printed using the SISMA MYSINT100 3D metal printer laser (Piovene Rocchette, Italy).

Sandblasting

The metal surface was sandblasted with 50-μm-sized aluminum oxide grains under a pressure of 0.7 MPa.

Testing the try-in metal frame

After obtaining the metal frame after the standard slot-back dummy was created, it was tested to achieve a single insertion line without any blocks, achieving the perfect fit for each wing into its designated groove designated for each of them, with complete stability in the gingival-occlusal and mesiodistal directions. This testing ensured that the dummy would not come out unless a force was applied from the buccal to the lingual side according to its insertion line.

Cementation

After preparing the samples, the adhesive bridges were adhered as follows. The retention parts of the metal prosthesis were cleaned by applying 10% hydrofluoric acid for 30 seconds, washing with water and air for 20 seconds, and then drying with dry air for 10 seconds, after which saline was applied.

The abutments were etched with 37% phosphoric acid for 30 seconds for the enamel and 15 seconds for the dentin. The abutments were then washed with water and an airstream for 15 seconds and then dried with a weak airstream for 10 seconds.

**Figure 7:**
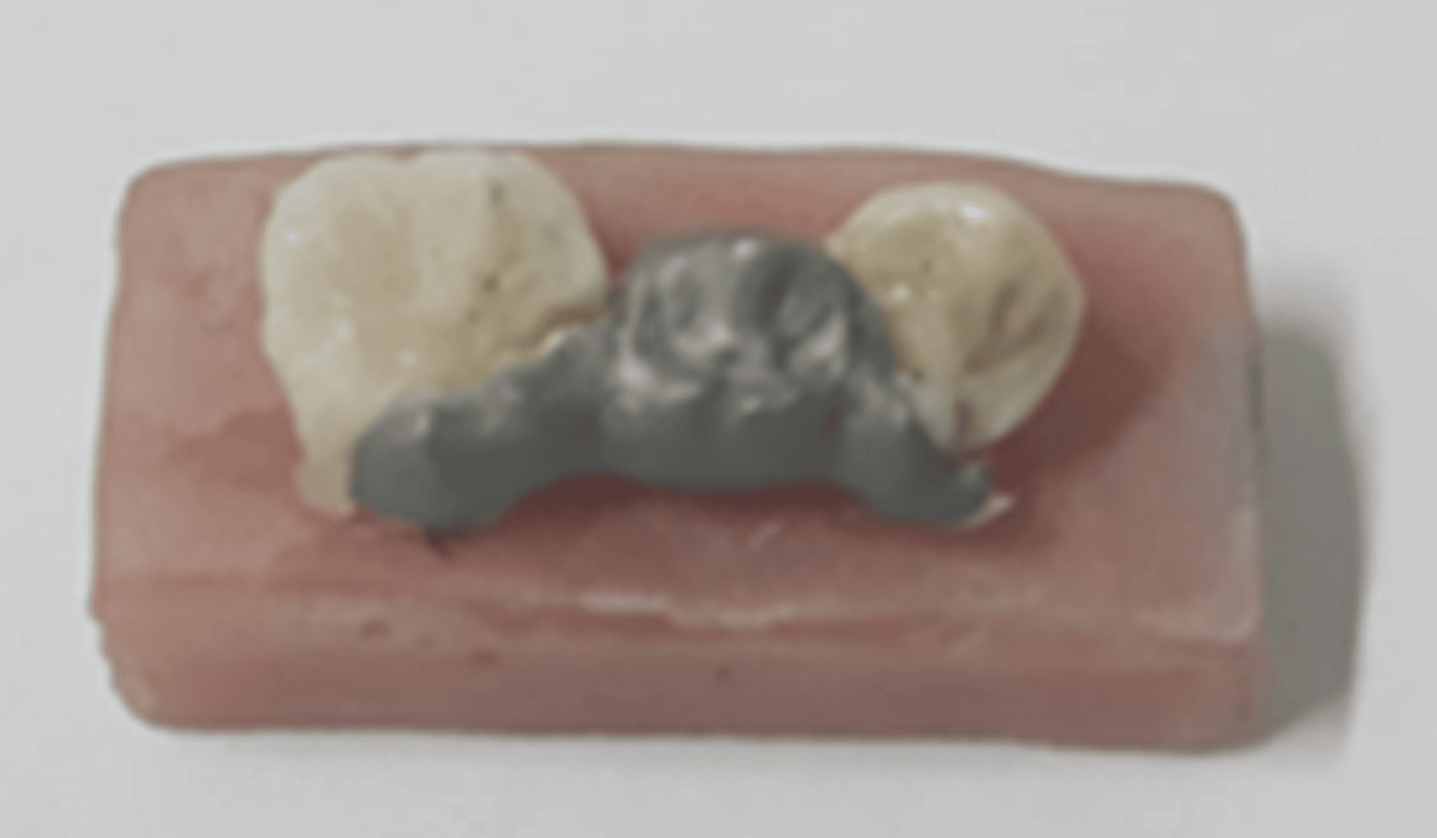
Traditional adhesive bridge after adhering

Next, a layer of Harvard bond was applied with a light airstream to distribute the bond on the surface; then, the double-curing resin (photochemical) was applied using special mixing heads. After the bridge was set in place, excess cement was removed with a fine brush and then cured with a light curing device for 60 seconds according to the manufacturer's instructions (Figure 7).

The same process was done to adhere all the specimens.

Mechanical testing

**Figure 8:**
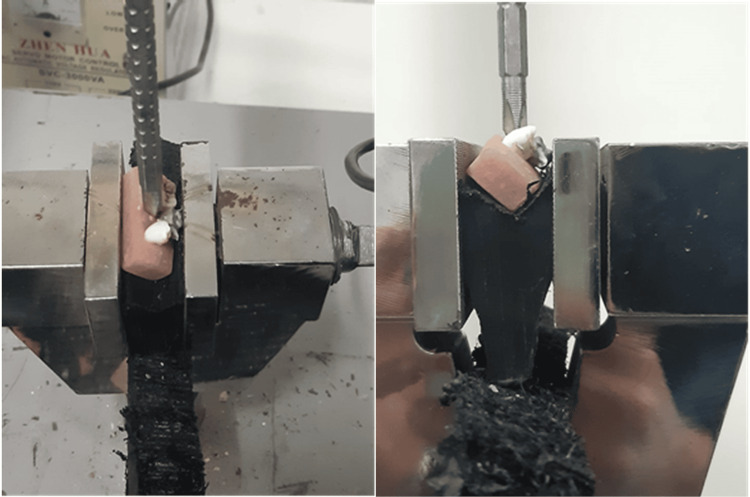
Forces applied at 45° using a universal mechanical testing device

Forces were applied to the samples using a universal mechanical testing device, which applied the force perpendicular to the sample using a pointed head for the force to be applied at an inclination of 45°. A base was made to support the sample at an angle of inclination of 45° as shown in Figure 8, and thus, the force is directed at the required inclination.

Statistical study

Statistical study was conducted using SPSS software version 24 (IBM Corp., Armonk, NY), and the one-way analysis of variance (ANOVA) test was used to study the significance of the differences in the resistance to bridge dislodgement across the three groups. The Bonferroni test was also used to study binary differences between the groups. The Microsoft Excel program (Microsoft Corp., Redmond, WA) was also used to illustrate the results reached in graphical forms.

## Results

Sample description

The research sample consisted of 36 bridges divided into three groups of 12, according to the method used in their preparation. Table 1 shows the distribution of the research sample according to the preparation method used.

**Table 1:** Sample distribution

Groups	Number/group	Percentage
Traditional adhesive bridges	12	33.3%
Slot-back dummy	12	33.3%
Modified slot-back dummy	12	33.3%
Total	36	100%

Studying the differences in the resistance to bridge detachment between the research sample groups

To compare the resistance to bridge dislodgement between the groups, a one-way ANOVA test was used; the results are shown in Table 2.

**Table 2:** Descriptive statistics of bridge dislodgement resistance values in the study groups

Groups	Number/group	Average	Standard deviation	Standard error	Minimum	Maximum
Traditional adhesive bridges	12	480.858	163.221	47.118	255.8	774.8
Slot-back dummy	12	486.050	175.625	50.699	287.7	902.9
Modified slot-back dummy	12	746.733	342.465	98.861	438.5	1372

The results in Table 3 show that the value of the one-way ANOVA test used to study differences in average bridge dislodgement resistance measurements between the three groups was 4.761; its p-value was 0.015, which is below the significance level of 0.05. Therefore, there are statistically significant differences in the average bridge dislodgement resistance values ​​between the three sample groups. To reveal the direction of the statistically significant differences, the Bonferroni test for binary comparisons was used. The results are shown in Table 4.

**Table 3:** One-way ANOVA test results F: the mean square of the between-group divided by the mean square of the within-group, Sig: statistical significance, ANOVA: analysis of variance P < 0.05: Significant differences P ≥ 0.05: No significant differences

Source of variance	Sum of squares	Degrees of freedom	Mean square	F ratio	P-value	Sig.
Between groups	554689.077	32	277344.539	4.761	0.015	Significant differences found
Within group	1922443.806	33	58255.873			
Total	2477132.883	35				

**Table 4:** Bonferroni test results Sig: statistical significance P < 0.05: Significant differences P ≥ 0.05: No significant differences

Groups	Average	Standard deviation	Difference between averages	P-value	Sig.
Group 1	480.858	163.221	5.1917-	1.000	No significant differences
Group 2	486.050	175.625			
Group 1	40.858	163.221	-265.87	0.033	Significant differences found
Group 3	746.733	342.465			
Group 2	486.050	175.625	-260.683	0.037	Significant differences found
Group 3	746.733	342.465			

**Figure 9:**
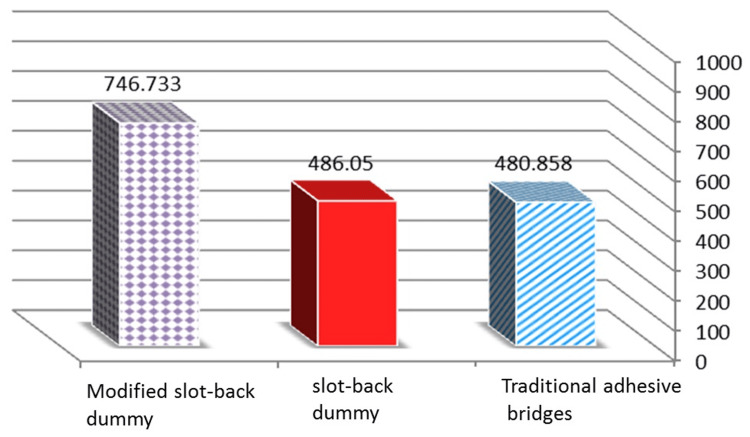
Differences between the averages of the bridge dislodgement resistance values between the groups X: groups, Y: the average of the bridge dislodgement resistance values

The results in Table 4 show that there is no statistically significant difference in the resistance to bridge dislodgement between the traditional adhesive bridge group and the standard slot-back dummy group. A statistically significant difference was found in the resistance to bridge dislodgement between the traditional adhesive bridge group and the modified slot-back dummy group in favor of the standard slot-back dummy group, which had an average measurement of bridge dislodgement resistance of 746.733 N, which exceeds the average of 480.858 N for the traditional adhesive bridge group (Figure 9).

In addition, there was a statistically significant difference in the resistance to bridge dislodgement between the group of the standard and modified slot-back dummy groups. This difference is in favor of the group of the modified slot-back dummy group, as the average resistance to bridge dislodgement was 746.733 N, higher than the average of 486.050 N for the standard slot-back group.

Study of failure modes

De-bonding of the prosthesis from the abutments, without fracturing either of them, was the failure mode recorded for the traditional adhesive bridges group and standard slot-back dummy group, while all abutments fractured in the modified slot-back dummy group (Table 5).

**Table 5:** Failure modes for each group N: newton

Groups	Average of the forces that caused the failure (N)	De-bonding	Prostheses fracture	Abutment fracture
Traditional adhesive bridges	480.858	12	0	0
Slot-back dummy	486.050	12	0	0
Modified slot-back dummy	746.733	0	0	12

## Discussion

While traditional bridge preparation consumes approximately 60% of the tooth structure, cavity size in the preparation of the slot-back dummy of the cavity does not exceed that of a class III restorative cavity; thus, it preserves the dental tissues well. Frequent de-bonding of adhesive bridges is also a common problem associated with this bridge type, which depends primarily on the adhesive cement for its stability. Despite the development of adhesive materials, this problem persists because the slot-back dummy design depends on both mechanical retention and adhesive retention. Furthermore, the location of the finishing lines is one of the problems we face in preparing traditional adhesive bridges, which are usually located at or very close to the gingival level, leading to recurrent gingivitis in cases of excessive margin length or improper preparation, while the preparation of the slot-back dummy is located above the gingival level, thus avoiding the bad relationship between the gingiva and the preparation margins.

The sample items were selected as mandibular second premolars were missing, and the abutments were mandibular first premolars and first molars to study different sizes of abutments. The sample size (N = 36) was selected consistent with the sample sizes of previous studies of the fracture resistance of minimal preparation prostheses [8,9].

The specimens were placed in acrylic bases to fix the extracted teeth that served as abutments and maintain an appropriate distance in accordance with the mesiodistal width of the lower second premolars. Metal alloys were used to manufacture the restorations to reduce the material cost since the study was conducted in vitro. Metal is also the preferred material for manufacturing these bridges because the structures in full-metal and metal-ceramic restorations provide higher durability and have simple re-bonding procedures in case the restoration becomes loose. In contrast, full-ceramic adhesive restorations are associated with high material costs and short durability due to fracturing of the ceramic, which is difficult to repair without replacing the prostheses [10].

A single-pack bonding system was chosen to minimize steps and because it demonstrated the best results in terms of resistance to shear forces. Selective acid etching technology was used to etch the enamel margins to ensure a strong bond with the margins of the restoration [11]. Sandblasting also increased the micromechanical bond of the prostheses.

Impressions were taken by CADstar Ultra digital scanner to avoid the errors of conventional impressions (impressions made with silicone or alginate do not give accurate results because the material in the retainer area tears when the impression is removed). This type of prosthesis also requires high accuracy to fit perfectly within the grooves. These bridges were designed using a computer to avoid waxing problems such as manual errors and metal shrinkage after casting; thus, the metal was printed using a SISMA MYSINT100 3D metal printer laser.

The 30° inclined angle was chosen in preparing the slot-back dummy to facilitate inserting the dummy, as greater inclination results in more difficult insertion.

The preparation was designed in compliance with the basic principles and conditions of preparing dental cavities: the entrance to the preparation in the dummy was from the lingual side in the buccal direction without exceeding the buccal surface, which achieves a higher aesthetic aspect and enables support from the buccal side.

The preparation corners were rounded to ensure a perfect fit for the restoration and to prevent concentration of effort at the preparation corners, using a 2-mm bur. In addition, the shapes of the burs in computer-aided manufacturing are rounded and do not give sharp corners. The entry point of the preparation bur was placed at the greater periphery of the tooth, so that the gingival wall of the preparation was at the level of the greater periphery, in order to create the largest possible support surface for the restoration, and to keep the preparation away from the pulp horn. The occlusal wall of the preparation was shorter than the gingival wall according to the anatomical shape of the tooth, and the unsupported enamel margins were beveled. The entry was achieved with the full diameter of the 2-mm bur to ensure sufficient thickness of the retainers to prevent fracturing or detaching from the dummy.

The arithmetic mean values ​​from the mechanical test ranged between 255 and 774 N in the traditional adhesive bridge group and between 287 and 902 N in the slot-back dummy group. These ranges can be explained by the differences in the contact surface area between the prosthesis and the tooth surface due to variations in tooth size and bonding strength between the enamel and dentin according to the degree of vitality of the abutment teeth due to differences in the extraction period between them. In addition, the thickness of the retainers differed in both designs.

Notably, the arithmetic mean values ​​were close between the traditional adhesive bridge group (480.858 N) and the slot-back dummy group (486.050 N), which may be because both designs achieved the retainer thickness estimated at 0.8 mm required to achieve the long-term success of these prosthetics [12]. In addition, both designs depended primarily on retention by cement bonding, as the preparation shape in the slot-back dummy group did not increase mechanical retention. The direction of the force applied to the prosthesis was also parallel to the removal axis of the prosthesis, which led to the de-bonding of the prosthesis from the abutments without fractures in either of them. In both groups, de-bonding is generally considered a mechanical complication that occurs for one of the following reasons: stress within the abutment, occlusal stresses, biomechanical properties of the prosthetic material, the quality of the bonding materials and their application technique, and failure stress [13]. The values ​​of de-bonding resistance in the modified slot-back dummy group ranged between 438 and 1372 N, and the arithmetic mean of these values ​​was higher than in the previous two designs (746.733 N). The difference between the first and second groups is due to the preparation angle of 30°, which made the angle of force application almost vertical; this force could not displace the restoration in the same direction as the axis of insertion and led to the failure pattern of abutment fracture. In other words, this difference in the results between the two previous groups is due to the shape of the preparation of the fixed abutments and not to the bonding force of the cement.

Several studies have evaluated the durability and clinical performance of adhesive bridges of different designs, and most agree with this study's finding that the main failure mode is de-bonding without damage to the abutments. It has been hypothesized that repeated tensile and compressive forces between the metal structure of the prosthesis and the bonding cement, with different degrees of individual movement in each abutment, are the main factors that lead to de-bonding [14-18].

The results of this study also agree with Mesbahi et al. (2018), who found that adding mechanical retainers in the form of inlays did not increase the stability or durability of the prosthesis, compared to traditional adhesive bridges with two wings as retainers; frequent de-bonding was a common complication of these prostheses [17]. In contrast, according to Albert et al. (2022), increasing the mechanical retainer types leads to an increased resistance of the prosthesis to shear forces. They studied the resistance of shear forces for three different designs: one included internal grooves (a grid) within the wing, the second included a mesh, and the third included a combination of the two other designs and showed the highest resistance [19]. The difference in results may be due to the increase in the number and shape of retainers in their study compared to the slot-back dummy design, in which the retention was limited to one upper cavity for each abutment; the difference in the direction of the applied force in the two studies may explain these different results.

The following factors are the main limitations of the study. First, the study was unable to control for the sample items of extracted teeth in terms of storage, anatomical shape, and the replacement of the first molar with the third molar in many samples. Second, this study was conducted in a dry environment and under static conditions that do not fully resemble the clinical situation. Third, oral forces differ from those in this in vitro study. Fourth, the clinical application of the study is difficult due to the need for a digital impression and thus the need for an intraoral scanner (i.e., higher cost) and the difficulty of its clinical preparation. Therefore, this study is considered a preliminary evaluation of the relative effectiveness of this bridge design, and this research must be supported by medium- and long-term clinical studies.

## Conclusions

Within the limitations of this study, adhesive bridges with modified slot-back dummies showed higher dislodgement resistance than the traditional adhesive bridges and the standard slot-back dummies.

In adhesive bridges with standard slot-back dummies, de-bonding without abutment damage was the failure mode, while the main failure mode in the adhesive bridges with modified slot-back dummies was abutment fracture.
